# Predicting self‐reported injury status among runners training for the New York City Marathon

**DOI:** 10.1002/pmrj.70127

**Published:** 2026-03-29

**Authors:** Mark A. Fontana, Jamie S. Egbert, Brett G. Toresdahl

**Affiliations:** ^1^ Orthopedic Data Innovation Lab Hospital for Special Surgery New York New York USA; ^2^ Department of Population Health Sciences Weill Cornell Medical College New York New York USA; ^3^ Department of Orthopaedics University of Utah Salt Lake City Utah USA; ^4^ Department of Physical Medicine & Rehabilitation University of Utah Salt Lake City Utah USA

## Abstract

**Background:**

Although numerous studies have examined risk factors and prevention strategies for running‐related injuries, few have rigorously tested predictive models.

**Objective:**

To describe injury patterns throughout marathon training and assess the feasibility of machine learning (ML) models in predicting upcoming weekly running injury status during marathon training with activity logs and runner‐reported survey data.

**Design:**

In this prospective observational study, participants completed baseline surveys, 16 weekly interval surveys during marathon training, and shared GPS watch and smartphone‐based running logs from Strava. Injury status was summarized and used to train two tabular ML models incorporating baseline, prior surveys, and aggregated training logs.

**Setting:**

All data were collected remotely via online platforms.

**Participants:**

A total of 643 adult runners (53% female, mean age 43 years) training for the 2022 Tata Consultancy Services New York City Marathon were recruited and had linkable Strava running logs.

**Interventions:**

N/A.

**Main outcome measure:**

Self‐reported weekly injury status indicating modification of training.

**Results:**

Of 643 runners, 307 (48%) experienced at least one injury requiring modification of training during the analysis window. Across 9002 runner‐week observations, 75% indicated no injury. Injury status tended to persist, with most runners maintaining the same status week‐to‐week, and recovery was more common than worsening. For the first model (all runner‐week observations), predictive modeling with generalized additive models yielded good performance, specifically an area under the receiver operating characteristic curve (AUROC) = 87% and area under the precision‐recall curve (AUPRC) = 52%. For the second model (previously uninjured runner‐week observations), performance was poor, yielding AUROC = 67% and AUPRC = 8%. Top features' partial dependency plots often showed nonlinear relationships with injury risk.

**Conclusions:**

ML using survey and running activity data had low discriminatory power in predicting weekly running injury among runners not already modifying their training, though results highlight the importance of the prior week's injury status. Larger samples, more precise injury timing, and additional predictors are likely needed to improve performance and inform future injury prevention strategies.

## INTRODUCTION

Marathon running has become increasingly popular over the last several decades.^1^, ^2^, ^3^, ^4^ Running‐related injuries are common and significantly affect long‐distance runners^5^, ^6^, ^7^ with injury incidence in general ranging from 19% to 79%.^6^ In retrospective surveys of marathon runners, the estimated training‐related injury incidence before a race ranged between 19% and 58%.^8^, ^9^, ^10^, ^11^, ^12^ This high injury burden results in ~1 in 10 marathon registrants being unable to reach the starting line.^13^

Despite the prevalence of studies on risk factors and potential prevention strategies for running‐related injury,^14^, ^15^, ^16^, ^17^, ^18^, ^19^, ^20^, ^21^ few properly tested predictive models exist for running‐related injury,^22^, ^23^, ^24^ and no intervention based on modifying training patterns with outputs from well‐validated predictive models has been shown to reduce injuries. Data from smartphones and GPS watches used to track running, containing rich information about running behavior that is passively collected and not biased by individual recall, have the potential to inform injury mitigation strategies and improve runners' health through predictive modeling. Predictive approaches are becoming more prevalent, spurred by excitement around artificial intelligence and machine learning (ML), that is, algorithms that automatically learn complex patterns and predictive relationships from data rather than relying on prespecified hypotheses.

The aim of the current study is to assess the feasibility of using ML models trained with smartphone‐based activity logs and runner‐reported survey data to predict self‐reported weekly running injury status indicating modification of training (ie, likelihood of injury indicating modification during the upcoming week of training given prior information). Although modeling efforts in previous work typically analyze all collected data in one analysis, purporting to uncover risk factors that are “predictive” of injury, the real test of predictive power is whether the model and associated risk factors perform well on different individuals than those used to build the model. Critically, we take this approach in evaluating our predictive models, advancing prior work^25^ by moving beyond common “association” analyses and evaluating predictive power on individuals not used to train our models. We also describe the severity of injuries during marathon training, including how runners transition from injury severity statuses week to week.

## METHODS

### Design

Runners consented to a prospective observational study administering pretraining baseline surveys and 16 weekly interval surveys among adult runners training for a marathon. We also asked runners to consent to sharing their GPS watch and smartphone‐based running logs from Strava (San Francisco, CA, USA). We trained tabular ML models to predict injury status indicating modification of training reported during the upcoming week using baseline surveys, prior interval surveys, and prior running logs. The study was approved by the first author's institutional review board on May 2, 2019 and extended on April 5, 2023 (Study ID 2019‐0279).

### Setting

The study was performed remotely with runners training for the 2022 Tata Consultancy Services New York City (NYC) Marathon (November 6, 2022). Runners were recruited by an email sent by New York Road Runners, the race organizer, to registered runners 18 weeks before the race (July 2, 2022).

### Participants

Eligibility criteria were: (1) age ≥ 18 years; (2) registered for the 2022 NYC Marathon; (3) U.S. resident; (4) not participating in another race that includes a 26.2+ mile run in the 16 weeks before the 2022 NYC Marathon and currently uninjured; (5) able to complete online surveys in English; and (6) willing to use Strava to track training runs. Altogether *N* = 894 runners registered, consented, and confirmed eligibility criteria. Runners were required to reconsent to share their training data with the investigators at the study conclusion. A data file containing all training logs from these runners was then provided by Strava to the investigators, yielding *N* = 643 reconsented runners with linkable training logs (643/894 = 72%).

### Variables

The primary outcome was running‐related injury in the upcoming week that resulted in modification or discontinuation of training (derived from weekly interval surveys). The definition of running‐related injury for this study, based on the consensus from Yamato et al.,^26^ was musculoskeletal pain that resulted in modification of running, including adjustments to training distance, speed, and duration, or discontinuation of training. Although the consensus also specifies a duration of modification (three sessions or 7 days) and considers health care consultation regardless of training modification status,^26^ we did not directly include these aspects into our injury definition. Reasons for this were (1) injury was measured on a weekly cadence with surveys and not triggered based on activity data, (2) our primary desire was to predict upcoming injury each week, and (3) this study was designed to capture self‐reported runner injuries and obtaining systematic clinical diagnoses during marathon training was not feasible. Instead, we assessed the degree of pain and extent of injury modification by adapting language from the Oslo Sports Trauma Research Centre Overuse Injury Questionnaire.^27^ Predictive factors (“features”) included variables from the baseline survey, prior weekly interval surveys, and prior weeks' Strava running logs.

### Data sources

Baseline and weekly interval survey data were collected using Research Electronic Data Capture.^28^, ^29^ Surveys were sent on Sunday each week (with two reminders), stopping after the study concluded or a runner stopped training.

Runners were instructed to track all running activities using Strava, an online platform and social network for tracking physical exercise. Training runs could be (1) recorded using the Strava mobile application on a GPS‐enabled smartphone; (2) recorded using a wearable device (eg, GPS watch) and synchronized with Strava; or (3) manually entered on the Strava mobile application/webpage.

The surveys and training data were linked and analyzed at the weekly level so that each runner contributed up to 16 observations. The marathon itself was not included, as our focus is on injury during training. Weekly interval surveys filled out >6 days after they were initially sent were treated as missing (1.6% of completed surveys).

### Statistical methods

We summarized runner baseline survey information with descriptive statistics and runner injury severity status with counts and percentages. These statuses describe the extent to which training was modified each week: no injury; injury with no modification; injury with modification to a minor/moderate/major extent; or stopped training (due to injury), stopped training (due to non‐injury), or not available. Injuries were classified as occurring from rapid onset of overuse injury, gradual onset of overuse injury, acute (traumatic) injury, other, don't know, or not available.

We also present a “transition matrix” illustrating changes in injury severity status from one week to the next, accumulated across all runners and pairs of weeks, reported as basic counts and row percentages (ie, given injury status one week, the number and percentage in each injury status the following week). This motivates our definition of “injured” for the predictive models, illustrates the importance of prior week's injury status, and motivates our two modeling specifications.

For the predictive analyses, our primary outcome was upcoming weekly running‐related injuries that resulted in modification or cessation of training, limited to those stemming from gradual or rapid onset injuries. Predictive factors (“features”) included 27 variables from: baseline survey (eg, demographics, running history), 1‐week prior interval surveys reported as of 1–7 days ago (eg, prior injury status, injury details, pain, fatigue, illness), and 2‐weeks prior training logs as of 8–14 days ago (eg, number of logged runs, weekly and monthly running distances and moving times, and 7:28‐day coupled acute‐chronic workload ratio based on total running distance [ACWR]) (Table 1). Table S1 contains exact survey questions for injury and predictors. We limited the timing of predictors to ensure that they do not unduly include information unknown before the injury happened. For example, at Week 2, it is possible that a reported injury occurred during Week 1 after that interval survey, so using training data measured during that week would be inappropriate, as it could include information from *after* the injury. Hence, we began our analyses at Week 3. Training logs for weeks among runners who explicitly indicated their logging was incomplete were set to missing (124/9002 = 1.3% of weekly training logs).

**TABLE 1:** Features (predictors).

Source	Timing	Feature	Description
Baseline survey	Pretraining, constant	rr_age	Age (continuous)
		rr_sex	Gender (male, female, prefer not to answer)
		bmi	Body mass index (kg/m^2^) (continuous)
		rr_orthotics	Orthotic/insert use (at least half the time) (Y/N)
		rr_happiness_b	General happiness in the last 7 days (1–6 Likert)
		rr_injury_prediction	Self‐perceived likelihood of training injury (0–100%)
		rr_running_hx_weekly_distance	Average weekly running distance (in 4 weeks before training period) (continuous)
		rr_running_hx_runs_per_week	Average days ran per week (in the 4 weeks before training period) (integer)
		rr_running_hx_marathon_completed	Number marathons completed (integer)
		rr_running_hx_previous_injury	History any running‐related injury in the past 12 months (Y/N)
N/A	Week	week	Indicator for week of training (3–16)
Interval survey	One week (1–7 days) ago	L_injury_modified	Injury status (−1 = not available, 0 = no injury, 1 = injury with no modification, 2 = injury with modification to a minor extent, 3 = moderate extent, 4 = major extent)
		L_how_injury_occured	How injury occurred (−1 = not available/no injury, 1 = acute injury, 2 = rapid onset injury, 3 = gradual onset injury, 4 = other, 5 = don't know)
		L_injury_location	Location of injury (−1 = not available/no injury, 1 = low back, 2 = hip, 3 = thigh, 4 = knee, 5 = leg, 6 = ankle, 7 = foot, 8 = other)
		L_fatigue	Fatigue in the past 7 days (1–5 Likert)
		L_happiness	General happiness in the past 7 days (1–6 Likert)
		L_illness	Illness status (Y/N)
		L_pain	Pain related to running in the past 7 days (0‐no pain, 1‐mild, 2‐moderate, 3‐severe)
Strava	Two weeks (8–14 days) ago	LL_week_num_activities	Number of logged runs (integer)
		LL_week_num_days_actvities	Number of days with runs (1–7)
		LL_week_day_max_distance	Maximum daily distance (continuous)
		LL_week_day_max_moving_time^a^	Maximum daily moving time (continuous)
		LL_week_tot_distance	Total 7‐day distance (continuous)
		LL_week_tot_moving_time^a^	Total 7‐day moving time (continuous)
		LL_month_tot_distance	Total 28‐day distance (continuous)
		LL_month_tot_moving_time^a^	Total 28‐day moving time (continuous)
		LL_tot_distance_acwr_4wk_div_mon	Acute: chronic workload ratio (4 × 7‐day distance/28‐day distance)

*Note*: See Table S1 for more details on exact survey questions.

Abbreviation: N/A, not applicable, as this was not data collected by survey or Strava.

^a^ Outliers (>2× 99th percentile) among weekly total moving time, weekly maximum daily moving time, and monthly total moving time were set to missing (likely inaccurate activity logs).

Two specifications were trained and tested varying the underlying sample: (1) all runner‐week observations and (2) runner‐week observations with *prior week* injury status of no injury or injury with no modification. The second focuses on runners not already modifying their training, which is “uninjured” according to our primary outcome definition.

In each specification, we randomly split the sample into training (80%) and test sets (20%) enforcing that all weekly observations from a runner were in one or another, that is, to prevent contamination of the test sets with information used in model training. We trained the models using the training sets and evaluated performance on the test sets using area under the receiver operating characteristic curve (AUROC) and area under the precision‐recall curve (AUPRC), including 95% confidence intervals (CI) by bootstrapping 1000 random iterations of the test set. Although AUROCs are commonly reported,^22^ they give credit for guessing “no injury” when this is likely to be correct. AUPRCs do not have this limitation and can be thought of as an average positive predictive value across probability‐of‐injury thresholds.

For our choice of ML algorithm, we leveraged a type of modernized generalized additive model (GAM) called an Explainable Boosting Machine, implemented with Python's *interpretml* package (v 0.2.7) (https://github.com/interpretml/interpret). These models learn the relationships between each predictor and the outcome in a data‐driven way, represented transparently and graphically, one graph for each predictor.^30^ Top predictors were measured and reported by rank‐ordering their mean absolute scores, which measure their absolute importance. If we had used linear or logistic regression, directional information would be learned and represented by the magnitude and signs of coefficients. GAMs instead learn *partial dependency plots*, one for each predictor, describing the full shape of the (possibly) nonlinear relationship between each predictor and the outcome graphically. This information is learned directly from the training data, with Explainable Boosting Machines harnessing shallow decision trees and boosting (iteratively improving weak models in sequence) to train the GAMs. Odds ratios (ORs) for injury risk can be calculated between any two values of a predictor by using the partial dependency plots, specifically by exponentiating the difference in *y*‐axis “score” (log odds) between any two *x*‐axis values. These models' nonparametric nature also allows us to set missing values of continuous predictors to arbitrary unused values (−1 here) instead of imputing, allowing comparison of risk among missing values alongside the full distribution of nonmissing values.

## RESULTS

Table 2 describes the baseline characteristics of the 643 participants. Throughout the analysis window spanning 14 weeks (after the first 2 weeks), 307 (47.7%) runners experienced at least one injury requiring modification of training, and 336 (52.3%) runners never experienced an injury requiring modification of training. Across the underlying 9002 runner‐week observations, weekly injury severity statuses were 75.1% for no injury; 5.5% for injury with no modification; 6.8% for injury with modification to a minor extent; 2.8% for moderate extent; 2.9% for major extent; 0.3% for stopped training (injury); 0.3% for stopped training (non‐injury); and 6.3% for not‐available (did not fill out an interval survey, filled it out late, or stopped training in an earlier week). These percentages do not dramatically vary across Weeks 3 to 16 (Table S2). A plurality of injuries was gradual onset of overuse injuries (40%–48% among injury statuses indicating some injury), followed by rapid onset of overuse injuries (18%–25%), and acute injuries (4%–10%), with the remaining of an unknown type (Table S3). The relatively low numbers of acute (traumatic) injuries motivated our focus on gradual and rapid‐onset overuse injuries beyond the intuition that acute injuries would be harder to predict.

**TABLE 2:** Baseline runner characteristics.

	*n* (%)
Total runners	643 (100%)
Age, years (mean ± SD^a^)	42.8 ± 11.9
Gender	
Male	299 (46.50%)
Female	343 (53.34%)
Prefer not to answer	1 (0.16%)
Body mass index, kg/m^2^ (mean ± SD)	23.9 ± 3.6
Orthotics or insoles in running shoes at least half of the time^a^	
No	485 (76.26%)
Yes	151 (23.74%)
General happiness^b^	
Extremely happy	63 (9.84%)
Very happy	281 (43.91%)
Moderately happy	268 (41.88%)
Moderately unhappy	23 (3.59%)
Very unhappy	5 (0.78%)
Self‐assessed likelihood of running injury during training assessed before the study^c^ (0–100%, mean ± SD)	41.12 ± 22.64
Percentage of participants with a history of running injury (that prevented running for at least 1 week) in the past 12 months before the study (%)	43.86%
Running distance/week (miles) in the month before the study (mean ± SD)	21.21 ± 13.27
Running days/week in the mo before the study (mean ± SD)	3.88 ± 1.45
Number of marathons completed (mean ± SD)	5.02 ± 6.06

^a^ Total sample size for this question was 636.

^b^ Total sample size for this question was 640.

^c^ Total sample size for this question was 600, from the survey question: “What do you think the likelihood is that you will experience a running injury while training for the marathon (0–100%)?”

Table 3 reports the transition matrix illustrating injury status in successive weeks. For example, among runners who reported no injury during one training week, during the next week 91.6% (*n* = 6228) reported no injury and 1.8% (*n* = 120) reported injury with no modification. Injury status tended to persist—injury status next week was *most* likely to be the same as injury status the previous week. Fortunately, more runners reported lower injury status next week (ie, recovery) than reported higher injury status (ie, worsening). These results highlight the importance of prior week's injury status. They also motivate our second modeling specification, which does not get “helped” by runners with existing injuries requiring modification of training, who are likely to continue having an injury requiring modification of training.

**TABLE 3:** Reported injury status from previous to next training week (transition matrix).

	Next training week								
Previous training week	No injury	No modification	Minor extent	Moderate extent	Major extent	Stopped training (injury)	Stopped training (noninjury)	N/A	Total
No injury	6228 (91.57)	120 (1.76)	170 (2.50)	66 (0.97)	54 (0.79)	3 (0.04)	20 (0.29)	140 (2.06)	6801 (100)
No modification	145 (29.41)	257 (52.13)	60 (12.17)	13 (2.64)	5 (1.01)	1 (0.20)	0 (0.00)	12 (2.43)	493 (100)
Minor extent	160 (26.53)	84 (13.93)	277 (45.94)	40 (6.63)	26 (4.31)	1 (0.17)	2 (0.33)	13 (2.16)	603 (100)
Moderate extent	56 (21.96)	8 (3.14)	67 (26.27)	81 (31.76)	32 (12.55)	4 (1.57)	1 (0.39)	6 (2.35)	255 (100)
Major extent	34 (12.50)	7 (2.57)	20 (7.35)	47 (17.28)	142 (52.21)	15 (5.51)	0 (0.00)	7 (2.57)	272 (100)
Stopped training (injury)	0 (0.00)	0 (0.00)	0 (0.00)	0 (0.00)	0 (0.00)	0 (0.00)	0 (0.00)	24 (100)	24 (100)
Stopped training (noninjury)	0 (0.00)	0 (0.00)	0 (0.00)	0 (0.00)	0 (0.00)	0 (0.00)	0 (0.00)	25 (100)	25 (100)
N/A	139 (26.28)	15 (2.84)	14 (2.65)	8 (1.51)	4 (0.76)	1 (0.19)	4 (0.76)	344 (65.03)	529 (100)
Total	6762 (75.12)	491 (5.45)	608 (6.75)	255 (2.83)	263 (2.92)	25 (0.28)	27 (0.30)	571 (6.34)	9002 (100)

*Note*: Cells contain sample sizes (top) and row percentages (bottom).

Abbreviation: N/A; not available

The first predictive model yielded an AUROC = 87.3% (95% CI: 84.2%–90.1%) and AUPRC = 52.1% (95% CI: 51.2%–52.4%) in the test set. The second predictive model yielded an AUROC = 67.3% (95% CI: 59.7%–75.5%) and AUPRC = 7.7% (95% CI: 6.6%–7.7%) (Table 4, Figure S1) in the test set. This decline in predictive power is the anticipated result of the “ease” of predicting that an injured runner is likely to remain injured.

**TABLE 4:** Model predictive performance.

	Model specification	
	All observations	Limit prior week injury status: “no injury” or “no modification”
# Observations^a^	8431	7142
# Features (predictors)	27	27
Training set # observations^a^	6739	5718
Training set # injuries	581	188
Training set % injured	8.6%	3.3%
Test set # observations^a^	1692	1424
Test set # injuries	177	50
Test set % injured	10.5%	3.5%
Test set AUROC^b^	87.3% [84.2%–90.1%]	67.3% [59.7%–75.5%]
Test set AUPRC^c^	52.1% [51.2%–52.4%]	7.7% [6.6%–7.7%]

^a^ Number of runner‐wk observations.

^b^ Area under the receiver operating characteristic curve [95% confidence interval].

^c^ Area under the precision‐recall curve [95% confidence interval].

Abbreviations: AUPRC, area under the precision‐recall curve; AUROC, area under the receiver operating characteristic curve.

**FIGURE 1:**
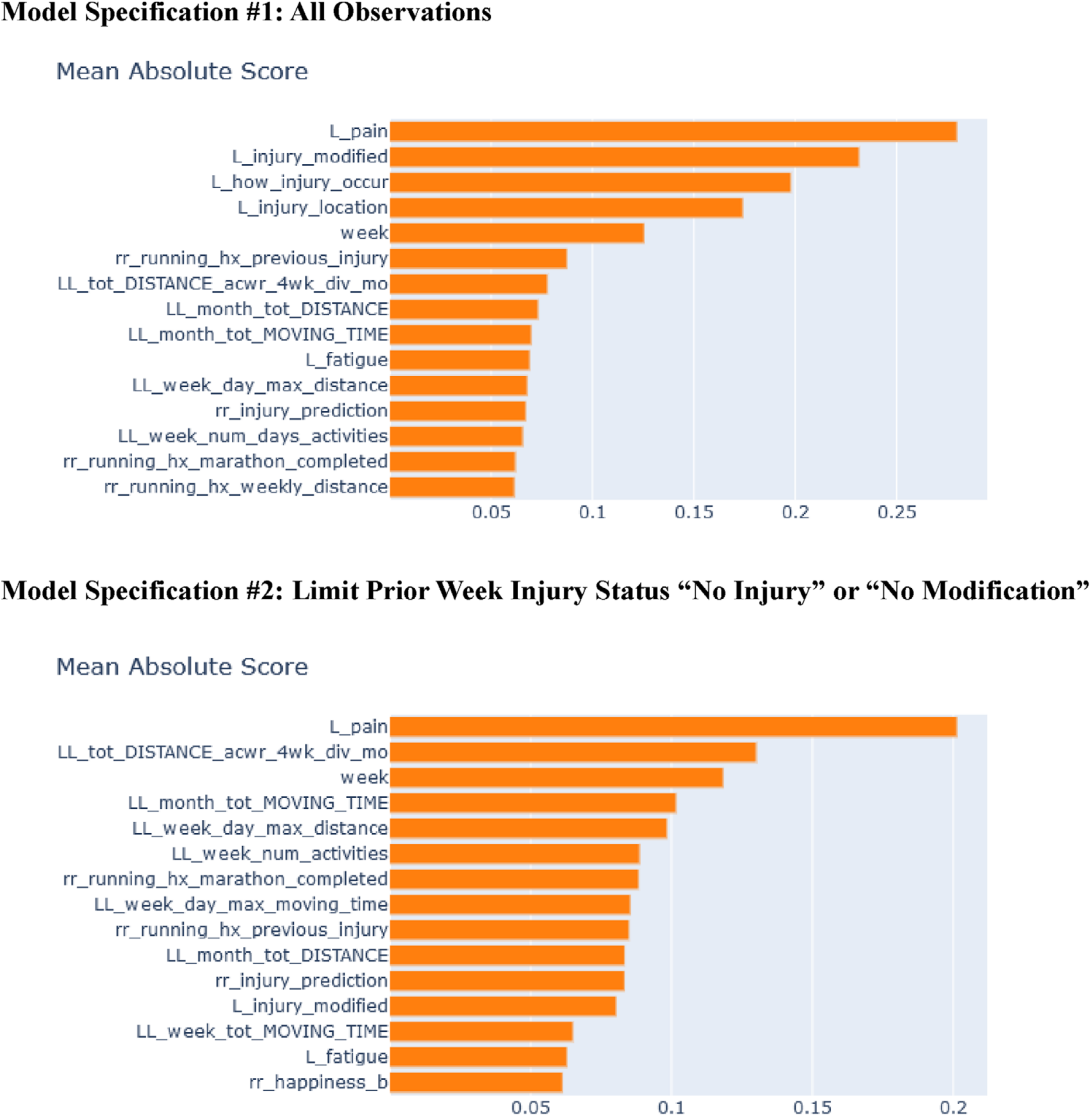
Top predictive features. Variable definitions can be found in Table 1.

Top six features for the first model were 1‐week prior reported (1) pain, (2) injury status, (3) how the injury occurred, (4) its anatomical location, (5) week of training, and (6) baseline history of running injury. For the second model the top six features were (1) 1‐week prior reported pain; (2) 2‐weeks prior 7:28‐day coupled ACWR; (3) week of training and 2 weeks prior, (4) total moving time in the past month, (5) maximum daily distance, and (6) number of logged runs (Figure 1).

Partial dependency plots for these top features reveal many nonlinearities (Figure S2). In the first model, higher prior week reported pain levels were associated with increased risk (eg, OR = e^(0.52 + 0.33)^ = 2.3 from moving from 0 = no pain to 2 = moderate pain). As anticipated, prior week injury severity status was also associated with increased risk (e.g., OR = 1.6 moving from 0 = no injury to 1 = no modification; OR = 2.8 moving from 0 = no injury to 3 = moderate extent or 4 = major extent). Risk was elevated for prior gradual and rapid onset injuries but not other types. It was also elevated for prior week reported injuries of the hip, thigh, knee, leg, ankle, foot, and other but not for low back injuries. Across weeks of training, risk increased at Week 7 (OR = 1.2) and again at Week 9 (OR = 1.2). History of running injury was also associated with increased risk.

In the second model, associations with prior week reported pain levels were similar (e.g., OR = 2.5 from moving from 0 = no pain to 2 = moderate pain) (Figure S2). ACWRs were nonlinearly associated, with risk at first flat, then increasing at ~1.3 (OR = 1.8), and finally flattening. Risk increased at Week 7 (OR = 1.3) and declined in the final weeks. Monthly moving times also showed a nonlinear association, at first increasing until ~500 minutes, then leveling off, decreasing after ~1000 minutes, and leveling off again after ~1700 minutes (peak to trough OR = 1.4). Weekly maximum daily distances were associated with high risk <3 miles; risk then decreased until 5 miles, increased again until 14 miles, and decreased slightly thereafter (peak to trough OR = 1.9). The number of weekly runs displayed a similar trend.

## DISCUSSION

Our aim was to assess the feasibility of whether ML algorithms could be trained with smartphone‐based activity logs and runner‐reported survey data to predict upcoming weekly running injury status indicating modification of training. We achieved good predictive performance in our first model using runners regardless of prior period injury status. However, for our second model, when we focused on runners without an existing injury requiring modification, predictive performance was much worse and driven more by training patterns. This is because this second model does not get “helped” by runners with existing injuries requiring modification of training, for whom it is likely that they will continue having such an injury requiring modification of training, which is relatively “easy” to predict. Critically, we evaluated predictive performance on runners that were not used to train the models, a distinction with implications for researchers that are discussed further below. Although model performance was not high enough to facilitate runner‐specific recommendations and injury prevention strategies, our generalized additive models allowed for a data‐driven approach that learned nonlinear associations between predictors and risk of injury with implications for advising runners. Our basic reporting of injury status over time, namely that runners typically sustain but trend toward recovery more than worsening, is also noteworthy. Although most injury prevention efforts focus on primary prevention, secondary prevention of injury is underappreciated yet also important for overuse injuries.

### Implications for research

The validation of predictive models is the first step before randomized intervention studies can be designed that curate their output for runners and test whether this information can spur modification of training and hence prevent injury. ML has been applied to injury prediction in other settings (eg, Australian football,^31^ Major League Baseball,^32^ National Hockey League,^33^ and soccer^34^, ^35^). Many are based on nonmodifiable factors; our current analysis includes, among others, training load, which has the potential to be modified. Here, performance—good among all runners, but poor among runners not already modifying their training—is likely a reflection of limitations on sample size and measuring exact injury timing. Although our results therefore indicate feasibility of this approach, performance was not high enough for designing runner‐specific injury prevention strategies, particularly for helping uninjured runners avoid future injury requiring training modification.

Many studies have used association frameworks (when all data are analyzed together and the focus is on identifying statistically significant associations with injury) to investigate risk factors for injury.^36^, ^37^, ^38^ These are often confused with predictive analyses that require testing the model with observations not used to build that model. For example, a prior iteration of this work from 2019 based on 725 runners collected *monthly* injury status over 4 months of training^25^ and hence did not have frequent enough injury data for prediction. In an association analysis using all data collected, the number of days in which ACWR exceeded 1.5 was associated with injury. Our current analysis treated ACWR as a continuous variable rather than dichotomizing it at predetermined thresholds, which has been subject to critique,^39^ finding a threshold at ~1.3.

Although there is a great deal of interest in deep learning algorithms that leverage high‐frequency sequence data or unstructured data such as images or free text, we opted to leverage more transparent, simpler models based on tabular data expressed at the weekly level and predict training modification from gradual‐ or rapid‐onset injuries. A scoping review from November 2024 of ML approaches to injury risk prediction in sport found only two papers about long‐distance running, with one relevant here.^22^ Lovdal et al. 2021,^23^ compared to us, studied fewer (*n* = 74), more experienced Dutch runners over a longer time frame (7 years) with fewer injuries (~1.4% fraction of injury events). They also leveraged a tabular ML approach and collected training logs with durations and distances, but unlike us, included information that would be difficult to collect on recreational runners (eg, exertion, training success, cross‐training). They reported a weekly specification comparable to our second model, reaching a comparable AUROC = 68% on a test set (*n* = 10 newest athletes) and a higher 78% on a randomized validation set. They also reported a daily specification that includes training information from the previous 7 days, performing slightly better in the test set, consistent with a benefit of including training data more proximal to injury. Using the same dataset, Ye et al.^40^ leveraged deep learning approaches, indicating benefits of more advanced methods, though at the cost of interpretability.

### Implications for advising runners

It is important to keep in mind that although the following implications are derived from broad associations learned by our modeling, for individual runners in our test set, these factors could *not* accurately predict injury among those not already modifying their training, warranting cautious interpretation as to their relevance for any given runner.

That said, our findings underscore the importance of current injury status for subsequent injury status, particularly gradual‐ or rapid‐onset overuse injuries. The information learned by our predictive models also highlights the importance of early recognition of an injury; pain was a top factor, and many runners who experienced pain that did not immediately result in training modification later developed an injury. Runners can be encouraged by clinicians, coaches, and race organizers not to ignore milder symptoms during marathon training that could lead to a more serious injury, which could benefit from targeted treatment or adjustment in training strategy in terms of total monthly moving time and weekly maximum daily mileage. Additionally, although changes in training load can be measured in various ways, an ACWR of 1.3 appears to be a relevant threshold under which runners should try to remain to reduce the risk of injury. Related, runners can also be reminded about injury risk increasing as the marathon nears (as shown by partial dependency plots indicating increased risk of injury after Week 7, ie, within ~2 months of the marathon). This timing is likely related to increased training loads, which are common at this point in training, and represent a time during which health promotion strategies could be especially prioritized.

### Limitations

This study has several limitations. First, sample size likely limited our predictive power, particularly among uninjured runners who became injured. This led to our focus on the feasibility of predicting rapid onset and gradual onset of overuse injuries, which were more common, but not acute injuries, which were rarer and likely harder to predict. Future data collection should endeavor to collect information on more runners, particularly to capture those transitioning from uninjured to injured states. Second, our sample of runners is likely not representative of those running the 2022 NYC Marathon nor marathons more broadly (eg, enrollment was more tilted toward female runners, Strava users are likely more technologically savvy, etc.). This may bias the relevant models and hence limit the external validity and generalizability of our conclusions. Selection bias from nonresponders to surveys and incomplete running logs could similarly impact the generalizability of our conclusions. Third, aligning the timing of weekly interval surveys with training logs was challenging, and for the sake of predictive fidelity, required using training logs from 8 to 14 days prior, but not within the last 7 days (hence starting prediction with Week 3). Future data collection should better measure the exact timing of injury to avoid this limitation. Relatedly, the numerous limitations of ACWR have been well‐documented, including statistical artifacts, lack of causal evidence, problems with calculating the initial load, and irrelevance during a taper before a race.^39^, ^41^, ^42^ That said, runners need a metric that is easy to track and calculate, and there are few straightforward alternatives. In this analysis, weeks 3–4 do not include a full prior month of training, and hence ACWR starts at 1.0 in Week 3 and does not account for a full month of training history until Week 5, which may bias the relevant partial dependency plots and affect performance. Given limitations in sample size and concerns about overfitting, we also considered training logs only from runs but not from other cross‐training activities; future, larger studies should consider such cross‐training activity. Moreover, injury status, completeness of weekly training logs, and other survey data were self‐reported by participants, which may affect their reliability given recall bias and other inaccuracies inherent to self‐reported data. Injury status also did not specifically ask about nonmusculoskeletal health problems, although we did ask about recent pain, illness, fatigue, and happiness. Finally, it is important to keep in mind that predictive modeling does not equate to causal inference, and our conclusions should be interpreted as such.

## CONCLUSIONS

Our study demonstrates the difficulty of predicting running‐related injuries during marathon training using training logs and runner‐reported survey data among those not already modifying their training. Our findings highlight the importance of researchers carefully considering the timing of injury and the need for approaches able to capture complex nonlinear relationships among training patterns, injury risk, and recovery. Our study contributes to the growing body of research on ML applications in sports medicine. Despite the low predictive power in our second specification, the information learned by our models has implications for sports medicine clinicians, subject to the limitations discussed here. Our findings also have implications for the future development of predictive models meant to be used to suggest modifications of training patterns and reduce running‐related injuries, acknowledging this is an ongoing, iterative process of refining data collection methods to improve the information used to train and test such models.

## FUNDING INFORMATION

Dr Fontana acknowledges support was provided by Schmidt Sciences, LLC.

## ETHICS STATEMENT

The study was approved by Hospital Special Surgery Institutional Review Board on May 2, 2019, amended on June 30, 2022, and extended on April 5, 2023 (Study ID 2019‐0279).

## DISCLOSURE

The authors have no competing interests to report. The results of the study are presented clearly, honestly, and without fabrication, falsification, or inappropriate data manipulation. In addition, the results of the present study do not constitute endorsement by the American College of Sports Medicine.

## PATIENT CONSENT STATEMENT

Informed consent was obtained from all participants.

## Supporting information


**Data S1.** Supporting Information.

## Data Availability

Research data are not shared.
