# Supplementary material for: Predicting self‐reported injury status among runners training for the New York City Marathon
Source: PM R. 2026 Mar 29;18(Suppl 2):S132–42. doi: 10.1002/pmrj.70127 (PMC13193517; doi:10.1002/pmrj.70127)

Supplemental Figure 2

Specification #1: All Observations

Feat #1

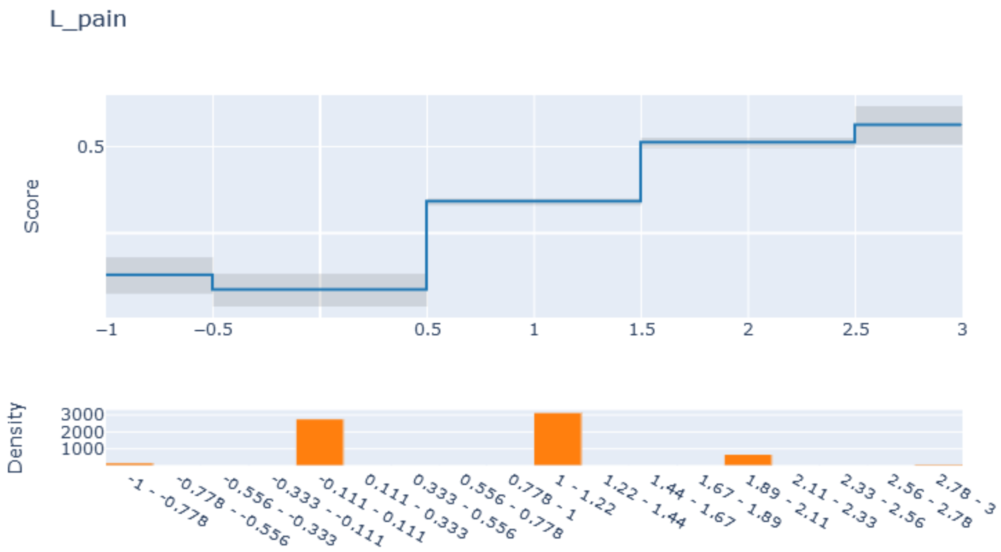

Feat #2

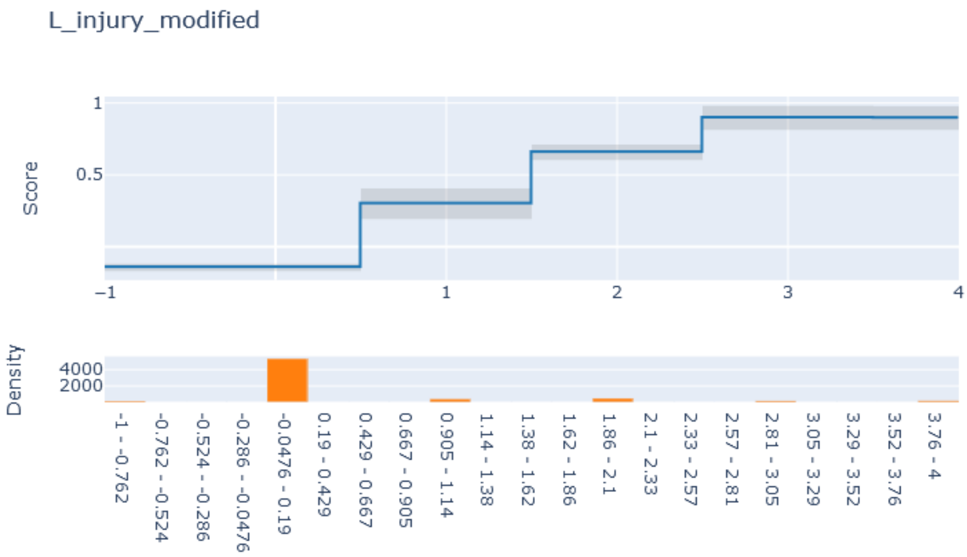

Feat #3

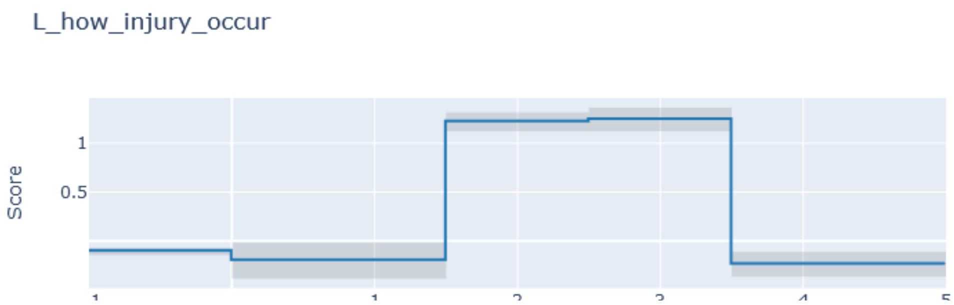

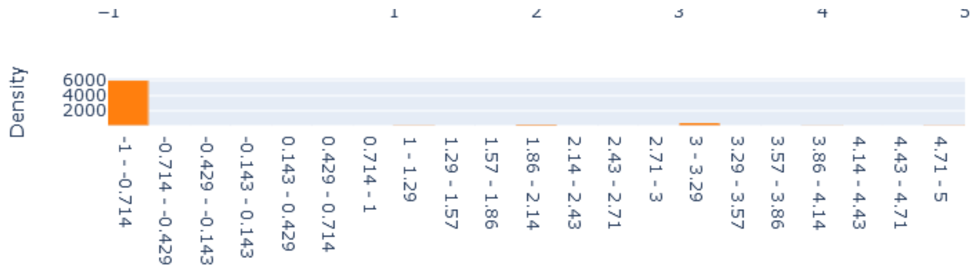

Feat #4

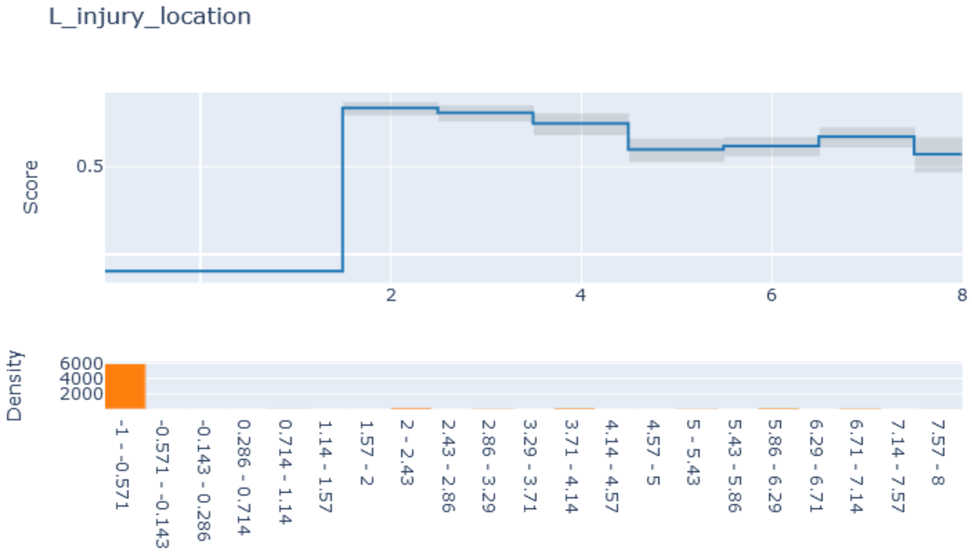

Feat #5

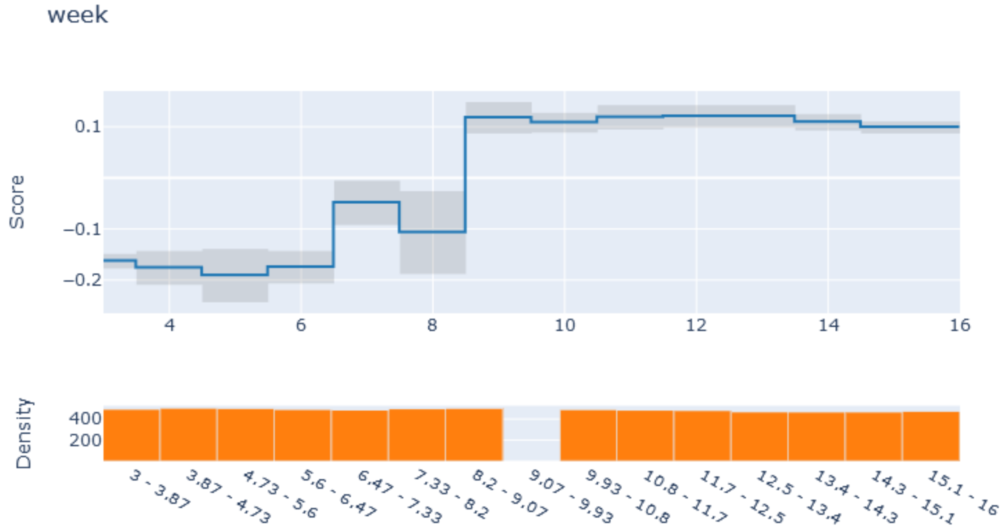

Note: Variable definitions and x-axis label meanings can be found in Table 1.

Specification #2: Limit Prior Week Injury Status “No Injury” or “No Modification”

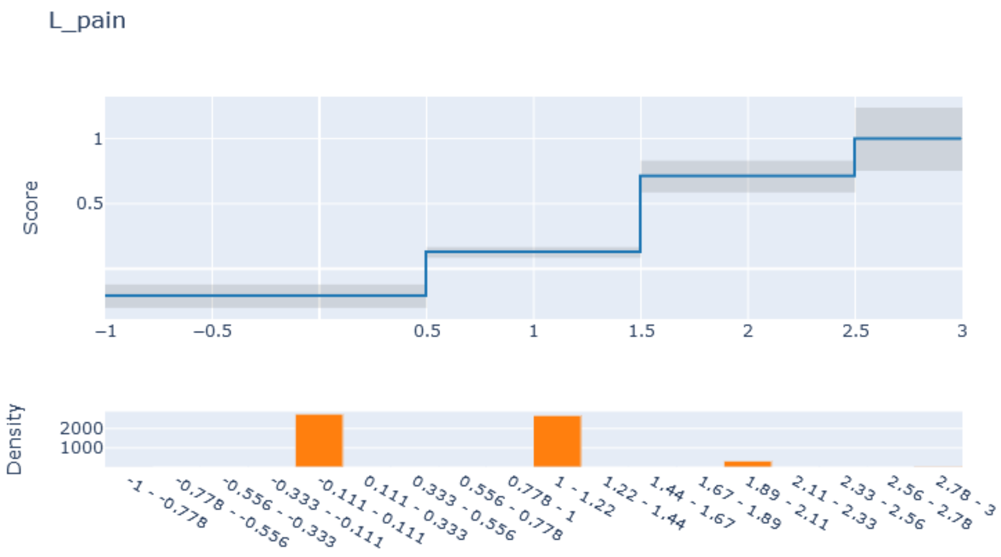

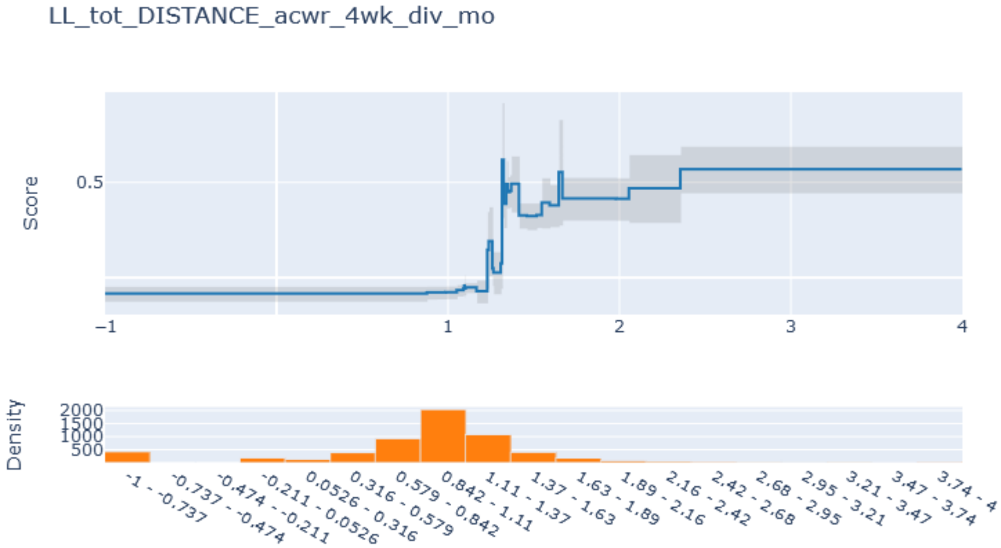

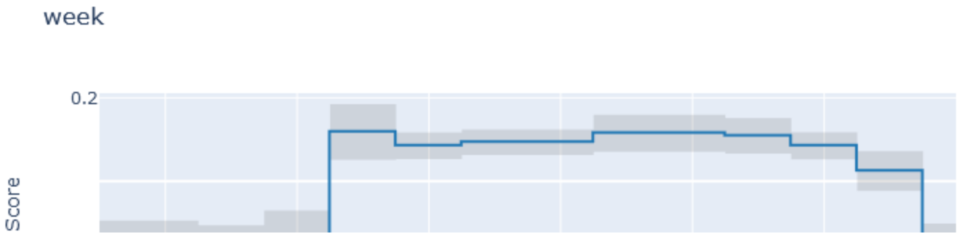

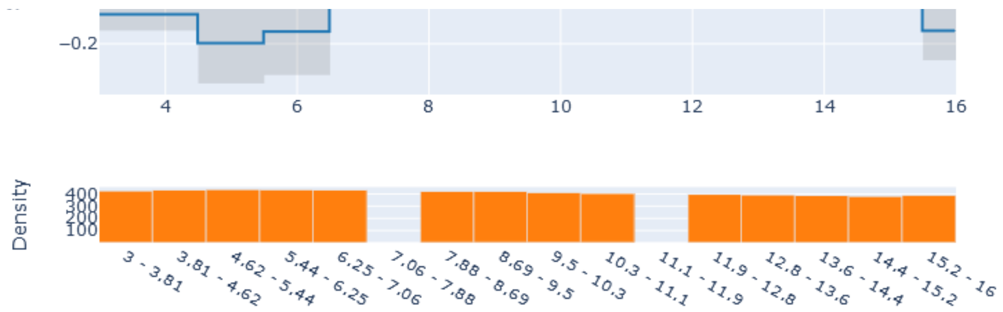

LL_month_tot_MOVING_TIME

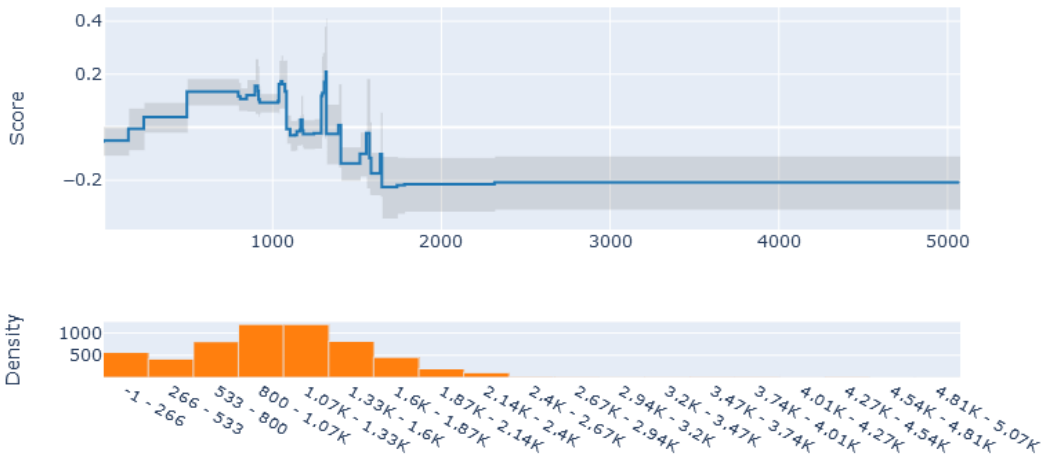

LL_week_day_max_distance

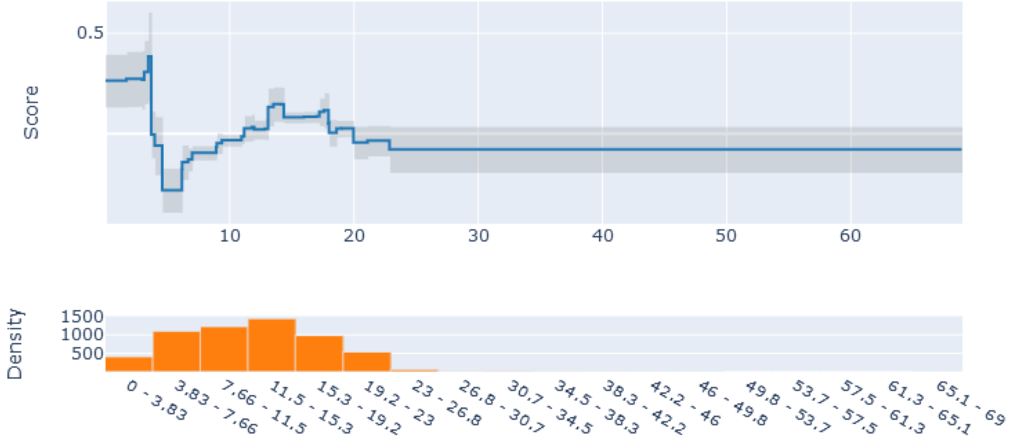

Supplement: Supplementary file 1 — Data S1. Supporting Information. [file PMRJ-18-S132-s001.pdf]
